# Label-Free Fluorescent Aptasensor for Ochratoxin—A Detection Based on CdTe Quantum Dots and (*N*-Methyl-4-pyridyl) Porphyrin

**DOI:** 10.3390/toxins11080447

**Published:** 2019-07-28

**Authors:** Li Liu, Zafar Iqbal Tanveer, Keqiu Jiang, Qingwen Huang, Jinghui Zhang, Yongjiang Wu, Zheng Han

**Affiliations:** 1 College of Pharmaceutical Sciences, Zhejiang University, Hangzhou 310058, China; 2 Institute for Agro-food Standards and Testing Technology, Shanghai Key Laboratory of Protected Horticultural Technology, Laboratory of Quality and Safety Risk Assessment for Agro-products (Shanghai), Ministry of Agriculture, Shanghai Academy of Agricultural Sciences, Shanghai 201403, China

**Keywords:** ochratoxin A, porphyrin, quantum dots, G-quadruplex aptamer

## Abstract

With the widespread contamination of ochratoxin A (OTA), it is of significant importance for detecting OTA in foods and traditional Chinese medicine (TCM). In this study, a novel label-free fluorescent aptasensor utilizing the interaction between OTA-triggered antiparallel G-quadruplex and (N-methyl-4-pyridy) porphyrin (TMPyP) for the rapid and sensitive determination of OTA was established. The fluorescence of CdTe quantum dots (QDs) could be quenched by TMPyP. In the presence of analyte (OTA), the aptamer could recognize OTA and transform from a random coil to the antiparallel G-quadruplex. The interaction between G-quadruplex and TMPyP could release CdTe QDs from TMPyP, and thus recover the fluorescence of CdTe QDs. Under optimized conditions, the detection limit of the designed aptasensor was 0.16 ng mL^−1^, with a linear range of 0.2 to 20 ng mL^−1^. Furthermore, this aptasensor showed high selectivity toward OTA against other structural analogs and other mycotoxins, and was successfully applied in *Astragalus membranaceus* samples. The presented aptasensor for OTA detection could be a promising tool for the field monitoring of food and TCM.

## 1. Introduction

Ochratoxin A (OTA) is a toxic metabolite derived mainly from *Aspergillus ochraceus* and *Penicillium verrucosum* [1,2], which are widely present as contaminants in a variety of products, including wines, corn, coffee, milk, and traditional Chinese medicine (TCM) [3,4]. OTA is found to cause severe toxic effects, such as those that are teratogenic, embryotoxic, genotoxic, neurotoxic, immunosuppressive, carcinogenic, and nephrotoxic to humans [5]. Since OTA presents a serious threat to the health and safety of humans, it was classified as being possibly carcinogenic to humans (Group 2B) by the International Agency for Research on Cancer (IARC) [6]. *Astragalus membranaceus,* as a major medicinal herb, has been broadly applied in clinical settings for treating diseases and promoting health [7]. It is especially important to ensure the safety of TCM, which has been frequently contaminated by mycotoxins during growth, collection, transportation, and storage [8,9].

Current analysis of OTA is conducted by various methods, including high-performance liquid chromatography (HPLC) [10], HPLC tandem mass spectrometry (HPLC-MS/MS) [11], and the enzyme-linked immunosorbent assay (ELISA) [12]. Although these methods have both high accuracy and sensitivity, they still have some shortcomings, such as being time consuming, and requiring tedious sample pretreatment and expensive instruments [13]. Biosensors are considered to be promising devices for the determination of chemical compounds such as toxins [14]. Among biosensors, the methods based on optical aptasensors are impressive [15]. Tian et al. have put forward a nanozyme-based cascade colorimetric aptasensor for the amplified detection of OTA [16]. However, the aptamer must be modified with fluorophores in these methods, while the manipulation of DNA modification is expensive and time consuming, which still urgently needs to be improved [17]. Therefore, it would be highly desirable to establish a rapid, convenient, low-cost, and sensitive method for OTA detection.

As an emerging type of fluorescent probe, the excellent optical properties of quantum dots (QDs) have meant that they have received tremendous attention in the fields of optical sensors, biosensing, biomedical analyses, and mycotoxin detection [18,19,20]. In the recent mycotoxin detection mode, the fluorescence change of quantum dots (QDs) was shown to be unidirectional, and the interaction with the analyte was evaluated by either quenching or enhancement [21]. However, this unidirectional mode may be affected by environmental stimuli and thus cause “false positive” results [22]. Porphyrins have been intensively reported as sensing materials in optical sensors [23]. They possess many attractive properties, including high chemical and thermal stability, high molar absorptivity, and a large extinction coefficient in the visible light region [24]. These optical advantages make porphyrin an ideal choice as the receptor in the photo-induced electron transfer (PET)-based “turn off” process. Recently, significant efforts have been made to apply such PET-based sensing platforms for the sensitive determination of various analytes such as DNA and metal ions [25,26]. However, as far as we know, no method has been reported for the sensitive detection of OTA utilizing (N-methyl-4-pyridy) porphyrin (TMPyP). Therefore, the unique aspect of this work is that we first report a label-free aptasensor detection method for OTA utilizing the interaction between TMPyP and OTA-triggered antiparallel G-quadruplex. The reported label-free fluorescent aptasensor has been successfully applied to the analysis of OTA in *Astragalus membranaceus*. It is expected that the aptasensor has tremendous potential for detecting OTA in TCM samples.

## 2. Results and Discussion

### 2.1. Design Strategy for OTA Detection

**Scheme 1:**
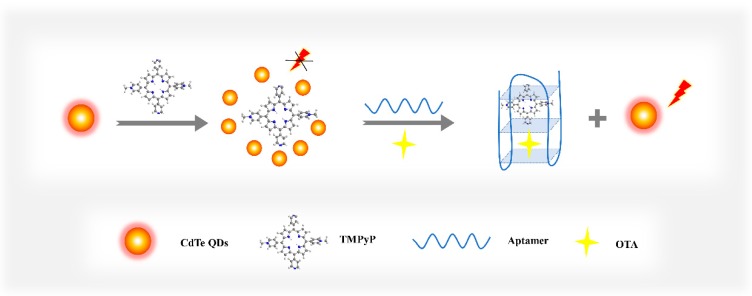
Schematic description of the fluorescent aptasensor for ochratoxin A (OTA) detection.

Scheme 1 shows the design strategy of a label-free fluorescent aptasensor for OTA detection. The fluorescence intensity of CdTe QDs was effectively quenched by TMPyP through PET [27]. In the presence of the analyte (OTA), the aptamers recognized the target and were induced to conformationally transform into the antiparallel G-quadruplex structure [28]. As TMPyP is a distinguished G-quadruplex ligand [29], it could interact with G-quadruplexes by outside groove binding and terminal π–π stacking interactions [30]. Since TMPyP occupied by G-quadruplexes was buried inside the structures, the interaction force between the CdTe QDs and the TMPyP became very weak, resulting in a significantly enhancement in the fluorescence intensity. Therefore, this restoration of fluorescence was interpreted as an analytical signal to evaluate the amount of OTA.

**Figure 1:**
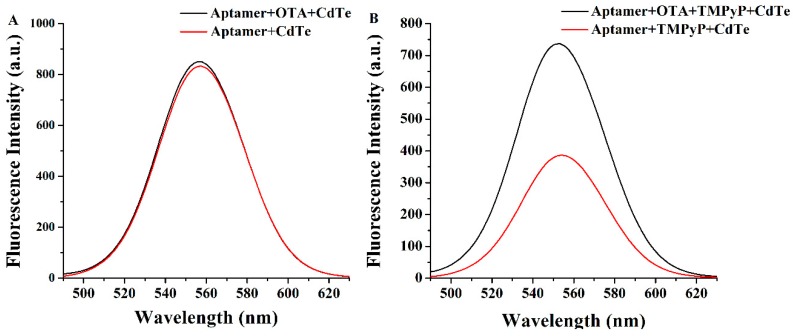
The fluorescence emission spectra of the CdTe quantum dots (QDs)/aptamer mixture with or without 20 ng mL^−1^ of OTA (**A**), and the fluorescence emission spectra of the CdTe QDs/aptamer/ (N-methyl-4-pyridy) porphyrin (TMPyP) mixture with or without 20 ng mL^−1^ OTA (**B**).

To further validate the feasibility of the established method, corresponding control experiments were carried out. In the absence of TMPyP, the fluorescence intensity of the CdTe QDs/aptamer was highly overlapping with or without OTA (Figure 1A). However, when TMPyP was included in the determination steps, the sample containing 20 ng mL^−1^ of OTA showed significantly higher fluorescence intensity than the blank sample (Figure 1B). This phenomenon proved that the fluorescence recovery of CdTe QDs was indeed caused by the combination of the TMPyP and G-quadruplex.

### 2.2. Characterization of G-Quadruplex Formation

**Figure 2:**
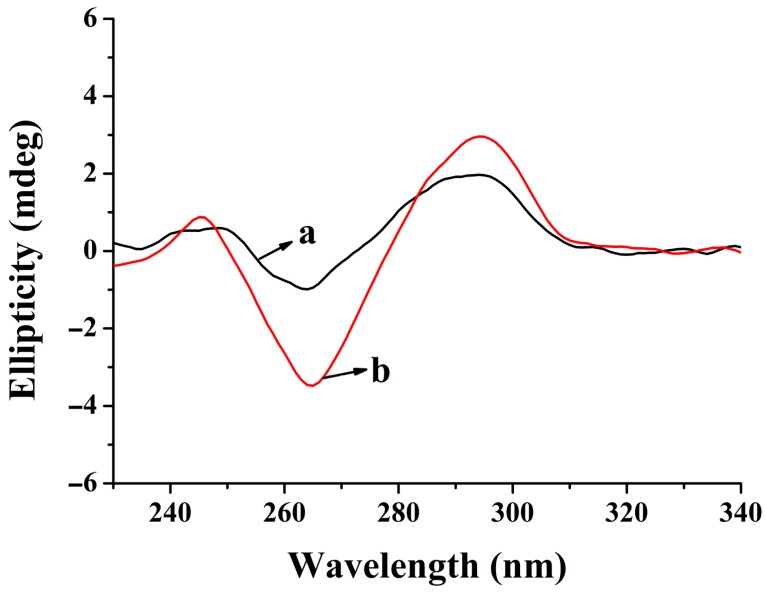
Circular dichroism (CD) spectra of the aptamer (5 μM) without (**a**) or with OTA (5 μg mL^−1^) (**b**) in 10-mM binding buffer (pH 8.0).

To verify the formation of the antiparallel G-quadruplex, the circular dichroism (CD) spectrum was employed. Before the introduction of OTA, the aptamer presented the characteristic of random-coil DNA (a in Figure 2). After the addition of OTA (5 μg mL^−1^), the CD spectrum exhibited an evident enhancement at about 265 nm for the negative band and at 290 nm for the positive band (b in Figure 2). This signal change in the CD spectra could be due to the interaction of OTA with the aptamer, which is a notable characteristic of antiparallel G-quadruplex formation [31].

### 2.3. Optimization of the Concentration of Porphyrin and Incubation Time

**Figure 3:**
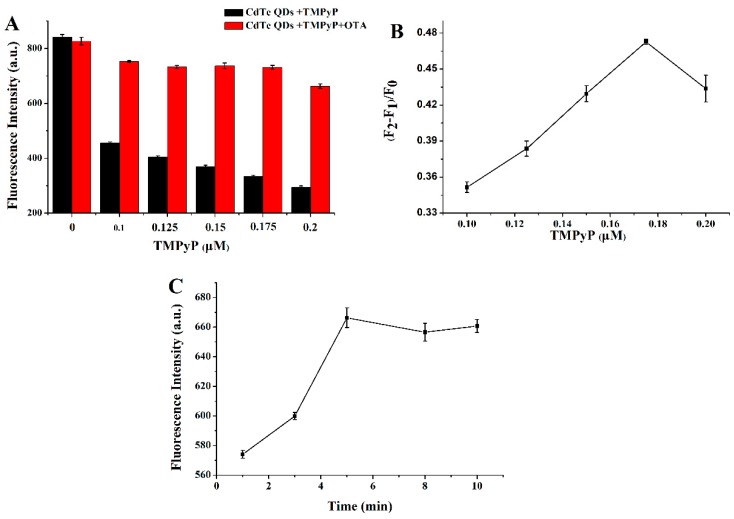
(**A**) Fluorescence intensity histogram of CdTe QDs/TMPyP and CdTe QDs/TMPyP/OTA aptamer in the presence of 0.1 μmol L^−1^, 0.125 μmol L^−1^, 0.15 μmol L^−1^, 0.175 μmol L^−1^, and 0.2 μmol L^−1^ of TMPyP. (CdTe: 4.2 × 10^−9^ mol L^−1^; OTA: 20 ng mL^−1^). (**B**) The linear plot of fluorescence intensity (F_2_ − F_1_)/F_0_ as a function of TMPyP (n = 3). (**C**) Fluorescence intensity as a function of incubation time (1, 3, 5, 8, and 10 min). (CdTe: 4.2 × 10^−9^ mol L^−1^; TMPyP: 0.175 μmol L^−1^; OTA: 15 ng mL^−1^).

To achieve high sensitivity for the label-free aptasensor, we optimized the concentration of TMPyP and incubation time. A higher concentration of TMPyP can largely quench the fluorescence intensity of CdTe QDs, and at a sufficient concentration of TMPyP, the fluorescence intensity of CdTe QDs can be completely quenched. However, an excess of quencher will inhibit the fluorescence recovery of CdTe QDs after the addition of an OTA aptamer, which is possibly due to the interaction between the G-quadruplex and “free” TMPyP. On the other hand, TMPyP deficiency does not effectively quench the fluorescence intensity of CdTe QDs, which may affect the detection of OTA. The value, I, was introduced to describe relative fluorescence recovery rates:

I = (F_2_ − F_1_)/F_0_ (1)

where F_0_ and F_1_ are the fluorescence intensity of CdTe QDs with and without TMPyP, respectively, and F_2_ represents the fluorescence intensity in the presence of the OTA aptamer. As shown in Figure 3, excessive or insufficient TMPyP is not advantageous to the fluorescence recovery of CdTe QDs. When the concentration of TMPyP was 0.175 μmol L^−1^, the fluorescence of QDs was quenched to 39.67%, and recovered to 86.90% of the original fluorescence of CdTe QDs after being exposed to OTA (20 ng mL^−1^). Based on this result, the concentration of TMPyP was fixed as 0.175 μmol L^−1^. As shown in Figure 3C, the incubation time was optimized, the fluorescence recovery reaches a plateau at 5 min. Therefore, the five minutes was chosen as the incubation time.

### 2.4. Quantitative Analysis of OTA

**Figure 4:**
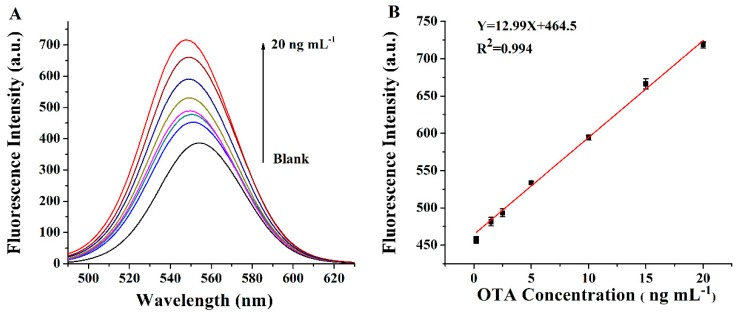
(**A**) Fluorescence spectra of the label-free aptasensor after the addition of different concentrations of OTA (0.2 ng mL^−1^, 1.5 ng mL^−1^, 2.5 ng mL^−1^, 5 ng mL^−1^, 10 ng mL^−1^, 15 ng mL^−1^, and 20 ng mL^−1^). (**B**) The linear plot of fluorescence intensity at 555 nm as a function of the OTA concentration (0.2 ng mL^−1^, 1.5 ng mL^−1^, 2.5 ng mL^−1^, 5 ng mL^−1^, 10 ng mL^−1^, 15 ng mL^−1^, and 20 ng mL^−1^) (n = 3).

Under the optimized conditions, we investigated the fluorescence spectrum after the addition of OTA. As shown in Figure 4A, a gradual increase in the fluorescence intensity at 555 nm was observed following an increase in OTA concentration from 0.2 to 20 ng mL^−1^. Figure 4B shows the linear relationship between the fluorescence intensity and the concentration of OTA (0.2 to 20 ng mL^−1^) (R^2^ = 0.994). The limit of detection (LOD) for OTA was calculated to be 0.16 ng mL^−1^ by three times the standard deviation of the background (3σ). As shown in Table 1, the linear range and LOD of the fluorescent aptasensor were compared with the current methods, and it was concluded that the current fluorescent aptasensor for the detection and quantification of OTA has a satisfactory linear dynamic range and detection limit. The repeatability was obtained by measuring the fluorescent intensity of the same sample (15 ng mL^−1^, OTA) six times, in which the coefficient of variation (CV) was 2% and the reproducibility was measured in different days, with a CV less than 2%, showing the good repeatability and reproducibility of this method.

### 2.5. Specificity of the OTA Aptasensor

**Figure 5:**
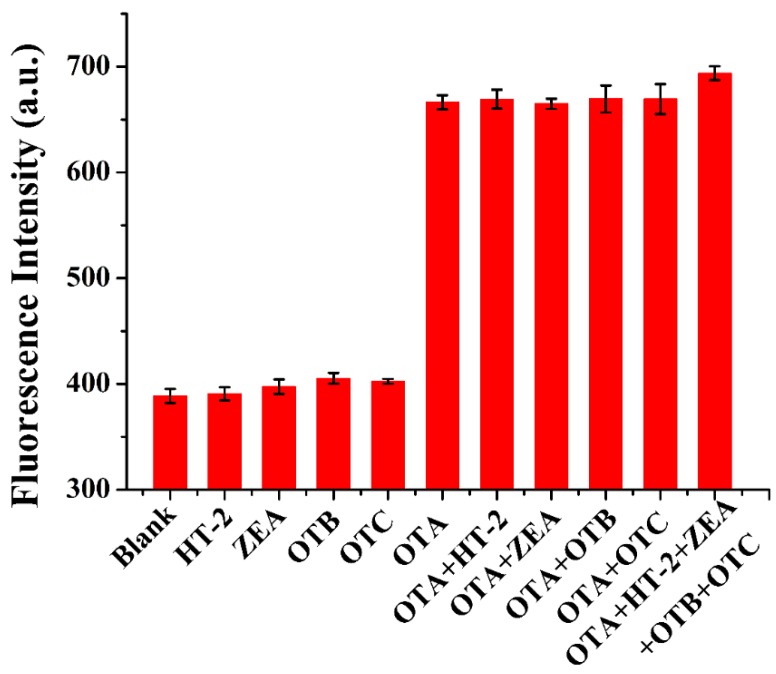
The specificity of the aptasensor toward OTA (15 ng mL^−1^) in the presence of its structural analogues and other mycotoxins (25 ng mL^−1^) (n = 3).

To confirm that the fluorescence intensity signal was caused by the unique recognition between aptamers and OTA, it was critical to test the specificity of the fluorescent aptasensor against other structure analogs of OTA (ochratoxin B (OTB) and ochratoxin C (OTC)) and other mycotoxins (zearalenone (ZEA) and HT-2 toxin (HT-2)). The concentration of OTA was 15 ng mL^−1^, whereas the concentration of the other toxins was 25 ng mL^−1^ in the specificity experiment. As shown in Figure 5, the fluorescence intensity displayed no evident change in the presence of HT-2, ZEN, OTB, and OTC. Considering that a sample may contain multiple mycotoxins, we also investigated the recognition performance of OTA in the presence of other mycotoxins. The signal responses did not change significantly when other mycotoxins were introduced to the aptasensor. These results clearly demonstrated that this fluorescent aptasensor for the determination of OTA exhibited an excellent specificity against different kinds of mycotoxins. The high specificity of the reported sensing platform was mainly due to the high affinity of the OTA aptamer. 

### 2.6. Detection of OTA in Real Samples

To evaluate the practicability and reliability of the presented aptasensor, we challenged our aptasensor with *Astragalus membranaceus* samples. As expressed in Table 2, the recoveries of OTA-spiked samples ranged from 98.9% to 102.2%, suggesting that this label-free aptasensor can be applied for the detection of OTA analysis in *Astragalus membranaceus*. However, CdTe QDs and the TMPyP based sensing system have limitations in detecting OTA in complex environmental samples, which may frequently contain nickel ions, Hg (II) ions, and pesticides. These coexistences can quench the fluorescence intensity of CdTe QDs and then affect the sensing ability of OTA detection.

## 3. Conclusions

In summary, a label-free fluorescent aptasensor based on the TMPyP/G-quadruplex for OTA detection was reported. Benefiting from a high specificity and affinity between the OTA and aptamer, the aptamer was switched into G-quadruplex, which could combine with TMPyP. This detection strategy avoids the laborious and expensive process of DNA modification. The designed sensing platform exhibits a superior range of detection and potential selectivity. The linear dynamic range and detection limits were 0.2 to 20 ng mL^−1^ and 0.16 ng mL^−1^, respectively. The application of the aptasensor has been verified in *Astragalus membranaceus,* with relative recovery values of 98.9% to 102.2%. This work provides a brand-new strategy for the OTA aptasensor based on the G-quadruplex-aptamer and TMPyP, and has great potential for applications for OTA analysis in food and TCM contamination detection.

## 4. Experimental

### 4.1. Materials and Reagents

Sodium tellurite (Na_2_TeO_3_), sodium borohydride (NaBH_4_), and cadmium chloride (CdCl_2_) were obtained from Aladdin Chemistry Co., Ltd. (Shanghai, China). Potassium chloride (KCl), magnesium chloride (MgCl_2_), calcium chloride (CaCl_2_), and acetonitrile were bought from Tianjin Yongda Chemical Reagent Co., Ltd. (Tianjin, China). N-acetyl-L-cysteine (NAC) was bought from Yuanye Biological Technology Co., Ltd. (Shanghai, China). TMPyP was chemically synthesized by J & K Scientific Ltd. (Shanghai, China). Hydrochloric acid (HCL) was purchased from Sinopharm Chemical Reagent Co., Ltd. (Shanghai, China). Tris [Tris-(hydroxy-methyl) aminomethane] was purchased from Shanghai Lingfeng Chemical Reagent Co., Ltd. (Jiangsu, China). OTA, OTB, OTC, ZEA, and HT-2 were purchased from Romer Labs Inc. (St. Louis, MO, USA) and dissolved in acetonitrile to prepare 10 μg mL^−1^ of stock solutions. The stock solutions were stored in the dark at −20 °C. The OTA aptamers (5′-GAT CGG GTG TGG GTG GCG TAA AGG GAG CAT CGG ACA-3′) [36] were synthesized and purified by Sangon Biotechnology Co. Ltd. (Shanghai, China). A binding buffer (10 mM of Tris-HCl, pH = 8.0, 120 mM of NaCl, 20 mM of CaCl_2_, and 20 mM of MgCl_2_) was prepared for the binding reaction between OTA and the aptamer. *Astragalus membranaceus* samples were purchased from Jiangxi Yaodutang Chinese Herbal Pieces Co., Ltd. (zhangshu, China).

### 4.2. Instrumentation

CD spectra were measured on a JASCO J-1500 spectropolarimeter (Tokyo, Japan) in 10 mM of binding buffer. The fluorescence spectra were acquired by a Hitachi F-2700 fluorescence spectrophotometer (Tokyo, Japan). The experimental parameters were set as follows: emission wavelength (λem), 490 to 630 nm; excitation wavelength (λex), 360 nm; emission slit, 10 nm; excitation slit, 10 nm. The deionized water used throughout the study was prepared by a Milli-Q Reference water purification system (Merck Millipore, Billerica, MA, USA).

### 4.3. Optimization of Experimental Conditions

In order to obtain the best experimental results, the concentration of TMPyP and incubation time were both optimized. The concentration range of TMPyP was 0.1 μmol L^−1^, 0.125 μmol L^−1^, 0.15 μmol L^−1^, 0.175 μmol L^−1^, and 0.2 μmol L^−1^. The fluorescence of CdTe QDs/TMPyP was performed as follows. Different concentrations of TMPyP were mixed with 80 μL of CdTe QDs (4.2 × 10^−9^ mol L^−1^) at room temperature. The fluorescence of the CdTe QDs/TMPyP/OTA aptamer was conducted as follows: 50 μL of OTA-aptamer (20 ng mL^−1^), different concentrations of TMPyP, and 80 μL of CdTe QDs (4.2 × 10^−9^ mol L^−1^). The incubation times were 1 min, 3 min, 5 min, 8 min, and 10 min. (CdTe: 4.2 × 10^−9^ mol L^−1^; TMPyP: 0.175 μmol L^−1^; OTA: 15 ng mL^−1^). The emission spectrum of the sample was measured, and each sample was measured three times.

### 4.4. Measurement of Aptamer Conformation with CD

The aptamer conformation was measured by a CD spectropolarimeter. The DNA aptamer (5 μM) and DNA aptamer (5 μM) with OTA (5 μg mL^−1^) were placed in an optical chamber (1-cm path length, 400 μL volume), which was deoxygenated with dry purified nitrogen (99.99%) prior to analysis and also insulated by a nitrogen atmosphere. The CD spectrum was run in triplicate with a 200-nm/min scan speed, 1-nm bandwidth, and 1-s time constant. The data were measured in a range of 230 to 340 nm at 0.1-nm intervals. The buffer solution was used as a blank to correct the background.

### 4.5. Determination of OTA by Fluorescence Aptasensors

For the quantitative determination of OTA, different concentrations of OTA were mixed with 250-nM aptamers in binding buffer and heated at 95 °C for 5 min, and were then cooled down to room temperature for upcoming experiments. After that, samples containing 175 μL of TMPyP (1.75 × 10^−7^ mol L^−1^), 80 μL of CdTe QDs (4.2 × 10^−9^ mol L^−1^), and 50 μL of OTA aptamer were made up to 1 mL. After equilibrating at room temperature for 5 min, the fluorescence emission spectra were measured at a wavelength range from 490 to 630 nm.

### 4.6. Specificity Assay

To verify the specificity of the aptasensor, HT-2, ZEA, OTB, and OTC were used as controls. Firstly, OTA (15 ng mL^−1^), HT-2 (25 ng mL^−1^), ZEA (25 ng mL^−1^), OTB (25 ng mL^−1^), and OTC (25 ng mL^−1^) were separately added into 250 nM of aptamer in binding buffer, and then heated at 95 °C for 5 min before being gradually cooled down to room temperature. Following this, samples containing 175 μL of TMPyP (1.75 × 10^−7^ mol L^−1^), 80 μL of CdTe QDs (4.2 × 10^−9^ mol L^−1^), and 50 μL of mycotoxins aptamer were made up to 1 mL. Multiple mycotoxins, including 175 μL of TMPyP, 80 μL of CdTe QDs, and 50 μL of OTA-aptamer, as well as other mycotoxins, were made up to 1 mL. After 5 min, the fluorescence intensity was measured.

### 4.7. Determination of OTA in Astragalus membranaceus

To verify the feasibility of this aptasensor, *Astragalus membranaceus* was chosen and finely grounded with a disintegrator. Then, samples were passed through a 0.22-mm aperture test sieve. A total of 1 g of the samples was spiked into different concentrations of OTA, and the mixtures were introduced into a 5-mL extracting solution (acetonitrile/water, 80:20, v/v). The samples were ultrasonicated for 10 min and then centrifuged at 4000 rpm for 10 min. The supernatant underwent a fivefold dilution with deionized water to minimize the matrix effect [37]. Additionally, subsequent manipulation was conducted according to the reported method.

## Figures and Tables

**Table 1:** Comparison of previously reported methods with the current study for the determination of OTA.

Method	LOD (ng mL^−1^)	Linear Range (ng mL^−1^)	Ref
Thioflavin T	0.4	1.2–200	[17]
SYBR Gold	6.7	8–202	[28]
Single-walled carbon nanohorn	7	8–202	[31]
QDs and MoS_2_	0.1	0.1–1000	[32]
Copper nanoparticles	0.8	1–100	[33]
PVP-coated graphene oxide	7.6	20–202	[34]
Single-walled carbon nanotubes	9.7	10–81	[35]
QDs and TMPyP	0.16	0.2–20	This work

**Table 2:** Application of the aptasensor for the determination of OTA in *Astragalus membranaceus* samples (n = 3).

Samples	Spiked (ng mL^−1^)	Detected (ng mL^−1^)	Recovery (%)
1	2.50	2.53 ± 0.19	101.3
2	10.00	9.88 ± 0.82	98.9
3	20.00	20.45 ± 0.83	102.2
